# Smartphone-based optical assays in the food safety field

**DOI:** 10.1016/j.trac.2020.115934

**Published:** 2020-08

**Authors:** J.L.D. Nelis, A.S. Tsagkaris, M.J. Dillon, J. Hajslova, C.T. Elliott

**Affiliations:** a Institute for Global Food Security, School of Biological Sciences, Queen's University, 19 Chlorine Gardens, Belfast, BT9 5DL, United Kingdom; b Department of Food Analysis and Nutrition, Faculty of Food and Biochemical Technology, University of Chemistry and Technology Prague, Technická 5, 166 28 Prague 6 – Dejvice, Prague, Czech Republic

**Keywords:** Smartphones, Colorimetric assay, Fluorescence assay, Food safety, Image analysis, Paper-based assay, Portable, Lateral flow immunoassay

## Abstract

Smartphone based devices (SBDs) have the potential to revolutionize food safety control by empowering citizens to perform screening tests. To achieve this, it is of paramount importance to understand current research efforts and identify key technology gaps. Therefore, a systematic review of optical SBDs in the food safety sector was performed. An overview of reviewed SBDs is given focusing on performance characteristics as well as image analysis procedures. The state-of-the-art on commercially available SBDs is also provided. This analysis revealed several important technology gaps, the most prominent of which are: (i) the need to reach a consensus regarding optimal image analysis, (ii) the need to assess the effect of measurement variation caused by using different smartphones and (iii) the need to standardize validation procedures to obtain robust data. Addressing these issues will drive the development of SBDs and potentially unlock their massive potential for citizen-based food control.

## Highlights

• Optical smartphone based sensors in the food safety field are systematically reviewed.
• Recommendations on image analysis optimization are given.
• The analytical performance of smartphone based sensors is discussed.
• Available commercial devises are critically compared.

## 1 Introduction

Easy-to-operate, portable and rapid devices have the potential to enable the execution of lengthy and complicated analytical chemistry protocols in the field, without the need for expensive equipment or high levels of expertise [1,2]. Using a smartphone as a processor and detector for this purpose is particularly attractive. Smartphones are ubiquitous; as such, hardware costs can be reduced significantly. Furthermore, the geolocation and internet connectivity of the smartphone can be used to pinpoint event location and ensure rapid data transfer to notify stakeholders in real-time. As a result, a plethora of developed smartphone based devices (SBDs) have been identified, showing applications in the medical, environmental and food security sectors [[3], [4], [5], [6], [7]]. Image analysis of colour-based assays (using the CMOS sensor and camera lens of the smartphone to photograph an assay) was by far the most commonly used technique for the SBDs discussed. This clearly indicates a preference for this approach.

Indeed, the simplicity of photographing an assay with the smartphone is attractive, especially if the amount of additional hardware needed to perform the measurements can be limited. Similarly, limiting measurement variation due to the use of different phone models is important. These issues are particularly pertinent to enabling food analysis with personal smartphones by consumers and primary producers. Thus, overcoming these issues in combination with further improving the technology readiness level of SBDs will be key to enable future use of SBDs by consumers. There are a myriad of smartphone models available, making it impractical to adjust every assay to every smartphone model. Moreover, consumers may be reluctant to adapt their smartphones with additional hardware to perform the analyses.

However, the development of appropriate SBDs can provide personalised food safety, which can cause a paradigm shift in the food sector whilst intensifying controls as individuals will be contributing to testing. Connected databases can be created and linked through apps to create a specialised, monitored and geolocalised warning system that is driven by citizen science. For example, such a system could communicate in real-time the presence of allergens in catering products, the unregulated use of pesticides in fruits, or contamination by *E. coli*, *Salmonella* or other pathogens. Consumers, producers, auditors and regulatory bodies could all find benefit in such systems. To make such a scenario possible, adequate sensitivity, reproducibility and ruggedness of the SBDs must be proven, and measurement variation among smartphones limited; fully integrated devices featuring multi-phone compatible software and hardware with a high level of technology readiness are required. Nevertheless, the analytical performance, the image data analysis strategies and the software and hardware use of food security SBDs have not yet been systematically compared. This makes it difficult to identify trends and critical technology gaps that need to be addressed to further the development of this field.

Thus, an in-depth analysis of current state-of-the-art SBDs for food contaminant and allergen detection has been performed here. Trends and critical technology gaps were identified and discussed alongside possible solutions. An overview of commercially available SBDs for food safety analysis is equally provided and critically reviewed. Moreover, various interesting alternatives that adhere to the basic idea of developing detection devices that are accessible to everyone were identified and discussed. The PRISMA (Preferred Reporting Items for Systematic reviews and Meta-Analyses) recommendations for the construction of a systematic review were followed to ensure that studies are selected in a systematic and unbiased manner [8].

## 2 Methods

This review used a structured keyword search and predefined inclusion criteria. Briefly, a keyword search was conducted in Scopus to identify optical SBDs in the food safety field. Only peer-reviewed experimental studies focusing on the food safety field were included in this review. Detection of the target analyte in matrix must have been done with the SBD for a study to be included. Initially 127 articles were identified after the keyword search. Of these, 56 met the inclusion criteria and were used to create a spreadsheet (supplementary table 1). This table lists the analytical parameters of the identified SBDs as well as critical information regarding the image analysis performed and the hardware and software used. A detailed description of the performed keyword search, selection process, exclusion (with reasons), and the parameters listed in the spreadsheet is given in the supplementary methods.

## 3 Analytical performance evaluation

### 3.1 Matrix complexity

**Fig. 1:**
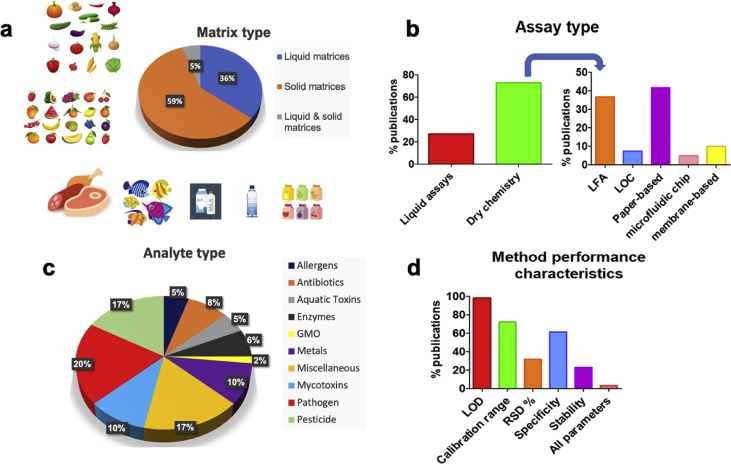
Studies classification based on the a) tested food matrix, b) assay and c) analyte type. d) Depiction of reviewed SBDs studies including method performance characteristics.

Of the 127 studies identified only 44% reported applications in a food matrix (including water), indicating a substantial bottleneck of colorimetric assays. Although considerable innovation related to devices architecture and sample handling integration has been achieved [9,10], this is not directly related to food analysis applications. Food analysis is difficult for many reasons; chief among them is the complexity of food matrices. Unlike water, food matrices are mostly solid and pigmented, as they are composed of various coloured components (e.g. chlorophylls, carotenoids etc.), and this can cause error in analysing the results from colorimetric assays. Thus, colour interferences may result in high limits of detection (LODs) or short linear ranges, a drawback when attempting quantification. Published data (Fig. 1a) indicates that about 14% of SBDs have been tested solely with water. Recently, some work has been done on the development of colour subtraction algorithms aimed at eliminating this problem but this work has as of yet only been validated for pH prediction [11].

### 3.2 Sample preparation

Sample preparation is another bottleneck preventing the wider adoption of colorimetric assays for food analysis. There are two reason for this:

(i) Recognition elements can be negatively affected by organic solvents (predominantly used to extract contaminants).
(ii) There can be a mismatch between the simplicity, portability and rapidness provided by colorimetric assays, and time-consuming sample preparation protocols.

To begin with, sample extraction makes it difficult to apply SBDs to solid samples, which may explain why about 30% of published studies target milk, fruit juice or honey (Fig. 1a). Laborious sample preparation protocols are commonly used, such as Easy, Cheap, Effective, Rugged and Safe (QuEChERS) method, which is the golden standard in multi-residue analysis [12]. QuEChERS employs acetonitrile as the extractant, a solvent able to extract analytes of various polarities, ranging from non-polar to semi polar. Another famous extraction protocol is the so called SweEt (Swedish Ethyl acetate) method [13], which uses ethyl acetate, a non-polar organic solvent, to extract pesticide residues from food matrices. In both cases, sample extraction may be followed by further clean up, using dispersive solid phase extraction (d-SPE). This is a convenient method in which sorbents (e.g. primary secondary amine or carbon black) are directly applied to extracts to remove undesired co-extracted compounds (for instance, organic acids or coloured pigments). This strategy may improve method trueness (depending on the selected sorbent, analyte and food matrix) [14]. Nevertheless, such laborious extraction protocols may hide colorimetric assay merits due to the time and expertise required. Thus, such sample preparation may be more appropriate for instrumental methods as they are more sensitive and robust but require more time.

As an alternative, simplified sample preparation protocols have been developed including sample homogenization, incubation in a buffer (during this incubation some protocols heat or shake the sample to increase extractability) and finally filtration to remove any solid particulate interfering matrix components [2]. An option that can potentially compensate the current challenging situation of complex extraction protocols is the development of micro total analysis systems (μTAS). μΤΑS provide integrated sample preparation, a highly-desired characteristic for in-situ and end-user friendly methods [15]. To date, there is a lack of such systems, especially in the case of solid food matrices. To develop μTAS the use of 3D-printing technology is highly recommended as cost-efficient, lightweight and practical solutions with short fabrication time can be developed [16].

### 3.3 Assay types

Notably, about 73% of published studies use dry chemistry assays, such as dipsticks with immobilized enzymes (e.g. acetylcholinesterase (AChE) or lipases) or lateral flow immunoassays (LFIA) (Fig. 1b). Such tests are simple (develop a colour response), rapid (<15 min) and portable (handheld) and results can be interpreted with a smartphone-based read-out. Firstly, to achieve long shelf-life, (i) various immobilization strategies [17], e.g. physical adsorption or covalent binding, and (ii) support materials, for instance nitrocellulose (exceptional protein binding ability) or cellulose (ubiquitous cheap material with capillary action), can be combined. The importance of support materials is also significant to the fluidic behaviour of the assay affecting response time or detection capability. With respect to enzyme assays, even if enzyme activity is inhibited by a group of analytes, for example AChE is inhibited by carbamate and organophosphate pesticides, or an enzyme is able to catalyse conversion of a group of analytes, e.g. diamine oxidase catalyses biogenic amines, the specificity profile highly varies depending on the analyte structure and enzyme source. This can be exploited to develop enzyme platforms capable to detect a group of analytes by performing cross-reactivity testing and select the most sensitive and specific recognition elements.

Multiplexing can be achieved by using wax-printing technology to create multiple immobilization spots on a membrane [18], which are efficiently separated by hydrophobic wax regions. Regarding LFA, great success has been achieved based on the WHO developed “ASSURED” principles, which stands for Affordable, Sensitive, Specific, User-friendly, Rapid and robust, Equipment-free and Deliverable to end-users [19]. Despite acquiring a “naked-eye” result when using LFA, smartphone readout can provide additional semi-quantitative results, allow rapid result communication and, in some cases, improve detectability. Although in some cases it was shown that the smartphone was less sensitive as the naked eye [20,21]. Further improvement of smartphone-based sensitivity may however be possible by adjusting shutter times and white balance (see section 4). To further enhance the optical detection of such assays, nanoparticles have been used as labels conjugated to antibodies and their aggregation on LF membranes was recorded by a smartphone [22]. Another interesting example was a handheld paper-based ELISA capable of detecting tetracycline at 0.050 ng.mL^−1^ [23]. This paper assay was successfully coupled to a smartphone app providing one-click results.

Besides dry chemistry assays, there have been also a few reports on smartphone-based liquid assays, mostly using a microplate format, which provides high throughput. Additionally, using a smartphone read-out for a microplate assay eliminates the need for benchtop spectrometers resulting in reduced analysis cost. This can be very useful for point of site detection in remote areas or in countries with limited resources. A striking example of using a smartphone instead of an absorbance reader is reported by K. Su et al. [24]. Here an optical system, was developed for marine toxins detection in shellfish achieving low LODs (Table 1). Nevertheless, smartphone-based sensing on microplate assays should be considered carefully during the development stage, as the microplate geometry can initiate multiple light reflections affecting the image quality. One possible solution to this is utilising a phone screen as a backlight source. This method was successfully applied in Ref. [25] for the quantification of gold nanoparticle solutions with an LOD ~ two to three fold lower as with a benchtop spectrometer while R^2^ values were >0.995.

**Table 1:** An overview of SBDs used in the food safety field focusing on their method performance characteristics. More publications can be found in the supplementary materials in spreadsheet form.

Type of analyte	Analyte	Matrix	Assay type	LOD	EU Legislative limits	Working range	RSD %	Specificity	Stability	Ref
Allergen	gluten, cow milk	milk, cheese	lateral flow devices, ELISA, nanoparticles on filterpaper	1.5 nM for colloid gold nanoparticles; 0.0025 nM for HRP	zero tolerance	colloid gold 3–13 nM, HRP 0.004–0.20 nM	NPs on filter paper: 25%–35% pH strips: 1%–2.5%	n.a.	n.a.	[25]
	hazelnuts, peanuts,	blank flour, peanut-spiked flour and different biscuits	LFIA and flow through using carbon black labelled antibody	1 and 10 ppm for hazelnut and peanut, respectively		n.a.	n.a.	2 proteins	n.a.	[21]
Antibiotic	streptomycin	honey, milk and tap water	AuNP aggregation (aptamer based stabilization) based on colour change	8.97 μg Kg^−1^	honey: restricted milk: 200 μg L^−1^	50–250 nM	0.7–8% depending matrix	5 interferent compounds	n.a.	[28]
	tetracycline	milk and honey	novel dye-doped porous metal–organic framework (UiO-66)-based	0.007 mg L^−1^	honey: restricted milk: 100 mg L^−1^	0.44–2.6 mg L^−1^	0.59–4.91%	various possible interferents	n.a.	[29]
Aquatic toxin	okadaic acid, saxitoxin	shellfish	cell viability in 96 micro-well plates	34 μg L^−1^	okadaic acid: 160 μg Kg^−1^ saxitoxin: 800 μg Kg^−1^	10–800 μg L^−1^	n.a.	saxitoxin and brevetoxin 3	n.a.	[30]
	okadaic acid, saxitoxin	mussel	indirect competitive immunoassay	0.09–0.009 ppb		0.02–0.32 ppb for STX; 0.2–5 ppb for OA	5–10%	cross reactivity study	n.a.	[24]
Enzyme	alkaline phosphatase activity (ALP)	milk	paper based assay catches ALP with mAb and substrate conversion	1.51 ± 0.17 UmL^−1^	n.a.	10–1000 U mL^−1^	<12% (in milk)	various interferents	28 days	[26]
	(l)-glutamate and other dehydrogenases	spice mixtures and bouillon	paper based enzymatic assay for colorimetric conversion	0.028 mM	n.a.	0.5–5.0 mM	<12%	various interferents	6 weeks	[27]
Metal	Cu	cucumber and tomato leaves, river water	paper based assay with Cu^2+^ chelating agent	0.795 μM	n.a.	10–2000 μM	n.a.	various ions	16 months	[31]
	Fe	meat and liver	chromogenic assays for iron ions	0.08 μg mL^−1^	n.a	depends on the colour space	n.a.	n.a.	n.a.	[32]
Miscellaneous	2-phenyl-phenol	water	sensory material developing a colour in the presence of a phenol	0.030 mg L^−1^	n.a.	n.a.	n.a.	metal cations and organic/inorganic anions	13 days	[33]
	formaldehyde	ginger, ginseng	Hantzsch reaction on mobile hotplate	0.2 ppm	n.a.	0.2–2.6 ppm	n.a.	n.a.	n.a.	[34]
Pathogen	*E.coli* and *Enterococcus species*	lettuce	enzymatic substrate conversion	0.2–2 μg ml^−1^	*E. coli:* 100–1000 cfu g^−1^ *Enterococcus species: n.a.*	0.1–1 mM	9–28%	n.a.	n.a.	[35]
	*Salmonella Enteritidis* and *E. coli*	milk and ice cream	nanozyme immunoassay in LFA format	~20 cfu mL^−1^ for S. Enteritidis and ~34 cfu mL^−1^ for *E. coli*	*Salmonella Enteritidis:* absence in 25 *g* *E. coli:* n.a.	n.a.	n.a.	various bacterial species	30 days	[36]
Pesticide	carbofuran	apple	hybrid LOC-AChE sensor	0.050 mg Kg^−1^	0.001 mg Kg^−1^	0.010–5.0 mg L^−1^	n.a.	n.a.	56 days	[16]
	chlorpyrifos, diazinon, and malathion	vegetables and fruits	fluoresent aptamer LFIA with diffraction grating and pixel to wavelength conversion	chlorpyrifos 0.73 ng mL^−1^, diazinon 6.7 ng mL^−1^, malathion 0.74 ng mL^−1^	chlorpyrifos: lowest possible diazinon & malathion: depending the matrix	n.a.	n.a.	atrazine, carbaryl, acetamiprid, and 2,4-D.	n.a.	[22]

AChE	acetylcholinesterase
ALP	alkaline phosphatase
HRP	horseradish peroxidase
LFIA	lateral flow immunoassay
LOC	lab-on-a-chip
mAb	monoclonal antibody
n.a.	not available
NPs	nanoparticles

### 3.4 Analyte types

SBDs are used to detect a wide variety of analytes, e.g. pathogens, pesticides, aquatic toxins or allergens. In fact, we distinguished 10 different analyte groups (Fig. 1c), with pathogens being the most common analyte (~20%), followed by pesticides (~18%), whilst metals and mycotoxins shared the third place (~11%). Among the groups, we classified one as “miscellaneous”, including, but not limited to: inorganic ions such as cyanide, preservatives such benzoic acid or dyes. Furthermore, based on analyte classification, Table 1 presents a summary of promising SBDs featuring both innovative assay set-ups as well as method performance characteristics whilst an additional number of reviewed studies can be found in the supplementary materials in spreadsheet tabulations.

### 3.5 Method performance characteristics

Regarding method performance characteristics, it was revealed that published research is mostly focused on reporting the assay's LOD (Fig. 1d). Although the detection capability of SBDs is a critical asset, as these methods are intended to be used for food safety regulatory control, other important characteristics, such as calibration range, specificity or repeatability also need to be evaluated. This finding is in accordance with our previous studies which indicated the problematic validation of screening assays in the food safety field [1,2]. Surprisingly, only two papers [26,27] studied all of the key validation parameters, namely LOD, calibration range, repeatability, specificity and stability (Table 1). Detection capability (CCβ) is a performance characteristic that can better define the detectability of screening assays as the LOD. However, to acquire CCβ it is necessary to perform a higher number of tests (both in blank and contaminated samples, commonly n = 20 for each case) compared to LOD, which is commonly calculated as the mean value of blank responses (usually n = 6) minus their standard deviation multiplied by 3. CCβ evaluates also method ruggedness, as measurements need to be performed in different days and in truly different blank and contaminated samples. Additionally, the false positive/negative rate can be defined by calculating CCβ, which is a critical parameter for smartphone-based assays. In the case of specificity, a characteristic of utmost importance to avoid false positive results, different approaches have been used. Some studies calculate the cross-reactivity rate whilst in other cases the analyte signal is measured in the presence of potentially interfering compounds. Overall, it is very clear that standardisation of the validation parameters when evaluating SBDs is highly needed to express their full potential in the food contaminant analysis (Fig. 1d).

## 4 Hardware/software development and image analysis procedures

### 4.1 Inter-phone variation

**Fig. 2:**
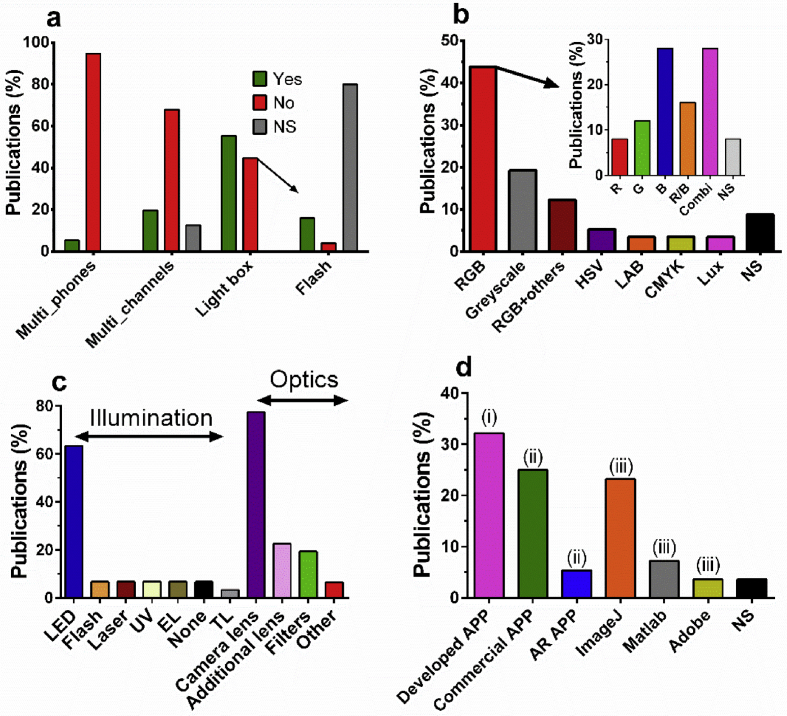
a) Percentage of analysed studies that compare assay performance with multiple phones, colour channels/spaces and percentage of analysed publications that use a light-shielding box or not. The percentage of publications that report using the phones' flashlight for illumination is given in respect to the reporting systems without light-shielding box. b) Proportion of colour spaces mentioned in the analysed publications. RGB + others indicates proportion of publications analysing RGB performance and at least one additional colour space. Lux indicates light intensity measured by the ambient light sensor of the smartphone instead of the CMOS camera. Inset proportions of the various colour channels utilised within RGB colour space. R/B means R and B channel used for different targets. Combi means various channel values were combined mathematically and related to the analytical signal. c) Proportions of various illumination sources and other optical hardware used to perform assays within a light-shielding box. d) Proportions of software used for data analysis, (i) SBDs with in-house developed software, (ii) SBDs that utilise existing colour apps, (iii) SBDs with off-phone data analysis using PC based software. NS is not specified. LED is Light-emitting diodes, EL is Electroluminescence, TL is tube light, UV is ultraviolet.

**Fig. 3:**
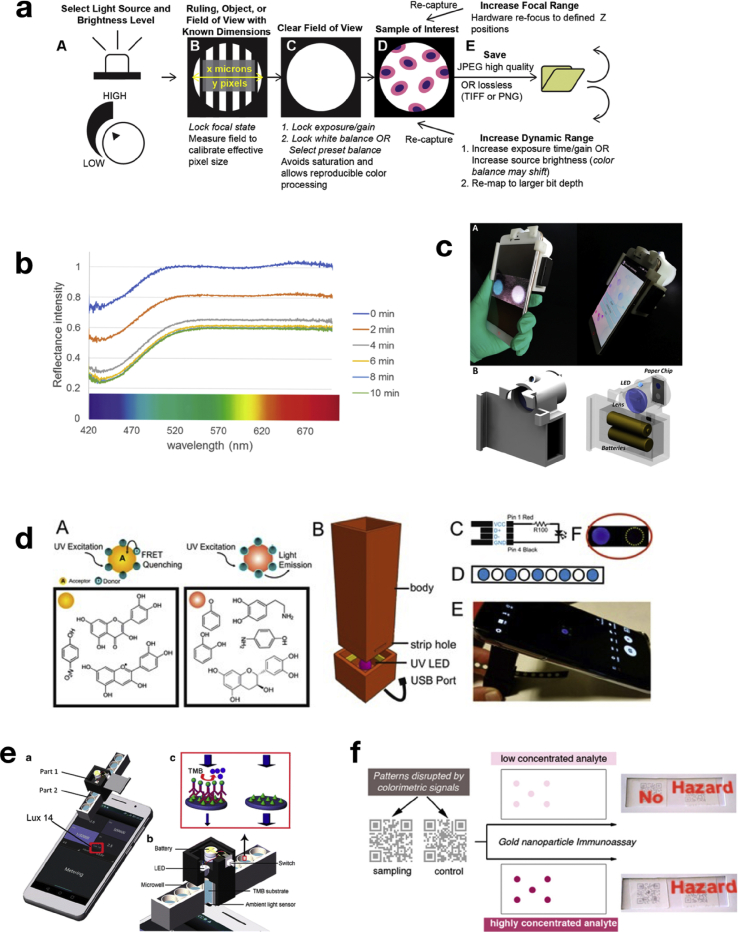
Examples of device configurations from papers described in the text. a) Suggested workflow to improve smartphone-based image quantification. Panel reprinted from Ref. [39] under CC BY 4.0 [54]. b) An example of measuring the spectral reflectance of the assay to determine in which region the analytical signal falls. Panel reprinted from Ref. [16] under CC BY 4.0 [54]. In this example, the response primarily modulates in the blue region. c) Cheng et al. developed a 3D printed fluorescent microscope to detect pesticides in vegetables and fruits, reprinted from Ref. [22] under CC BY 4.0 [54]. d) Ávarez-Diduk et al. have used UV excitable labels and UV-LEDS in a small and portable box that can be attached to the smartphone camera to measure polyphenols in wine samples. Panel reprinted from Ref. [47] under CC BY 4.0 [54]. e) Chen et al. have utilised the smartphone ALS to reduce error caused by background illumination and reflection for zearalenone detection in cornflower by ELISA. Reprinted with permission from Ref. [49]. f) An AR barcode SBD for *E. Coli* detection in drinking water, reprinted with permission from Ref. [52]. The assay is designed as a barcode, which is disturbed or changed by analyte recognition; the altered barcode is then read by a smartphone app which displays the final result.

The proportion of various image analysis tools, hardware and software used in the identified literature is given (Fig. 2). Of the 56 articles analysed only three used different smartphones to detect the effects of inter-phone variation on the measurements [21,25,37]. Ross et al.*,* used background corrected L-values from CieLAB colour space and showed good overlap of calibration curves constructed from carbon black labelled LFIAs and flow through assays for hazelnut and peanut detection (LOD 1 ppm for Hazelnut, 10 ppm for peanut; Table 1) [21]. However, only two smartphones were tested and variation on the predictions obtained with the various phones or the RSD were not calculated. Another study described quantification of sulphite in foodstuffs through blue intensity variation caused by sulphite driven Fe (III) reduction. The study reported no significant statistical differences in the predictions obtained with five smartphone models [37]. The study reported good sensitivity (LOD 0.04 μg.L^−1^) and correlation coefficients > 0.99. However, the calibration curves used for these predictions were built for each smartphone model individually and not visualised. The need to build calibration curves for each and every smartphone model is cumbersome and unrealistic for wider adoption, and thus, it should be avoided. In another study, six smartphones were used to quantify colour change (pH determination in soil using the R channel) and intensity-based assays (LFIA for gluten quantification). For pH determination, interphone variation on the predictions was less than 10% using one universal calibration curve for all phone models, however, individual calibration curves for the LFIA quantification did not overlap. This indicates that inter-phone variation without individual phone calibrations may be more limited in colour-change-based assays [25]. Another observation in this work is the substantial difference in the performance of various colour channels as well as the outstanding sensitivity of SBDs for quantification of liquid assays. For instance, for colloid gold nanoparticle quantification in wells the best performing channel was G (R^2^ = 0.9969; LOD 1.5 nM). For the B channel the R^2^ = 0.9973 and the LOD was 1.8 nM. The R channel could not be used to generate a calibration curve. Moreover, the performance of the L channel was significantly lower (R^2^ = 0.8947) while the LOD could not be calculated. For comparison, the curve generated with OD values from the benchtop spectrometer gave an R^2^ of 0.9996 and a LOD of 7.5 nM (thus three fold less sensitive as the smartphone when G channel was used) [25]. Some studies have developed methods to overcome the problems of inter-phone error variation such as by utilising a colour reference chart to correct colour variations caused by the built-in image correction operations [38] or by fixing the white balance, gain and exposure settings of the camera [39]. The image correction algorithm was used for pH predictions and utilised the provided reference charts from the manufacturer to calibrate the different cameras. This method showed little inter-phone variation (maximum difference in average error ± 0.2 pH units). However, large absolute errors ± 1.0 pH units) were observed for all tested phones for some pH predictions. These high error margins were attributed to differences between the printed colour chart and the actual assays, unequal LED illumination in the light-shielding box and colour saturation caused by automatic brightness control and white balancing [38]. The method proposed in Ref. [39] is especially appealing due to its simplicity. Moreover, it was shown that both Android and iPhones (various models) with a >5 MP resolution were capable of nearly diffraction limited resolution while inter-phone variation was limited significantly by simply locking camera settings using commercially available camera apps [39]. Moreover, the authors showed that gamma decoding (using an exponent of 2.2) of the pixel response can greatly improve linearity between intensity and pixel response. In fact, the authors were able to recover a linear pixel response of R^2^ = 0.999 from an initially non-linear correlation. The suggested workflow to improve smartphone-based image quantification while limiting inter-phone variation is reproduced in Fig. 3a.

### 4.2 RGB colour channel choices

Interestingly, the choice of colour space/channel used is not always empirically determined; colour spaces/channels tend to be chosen based on the colour of the assay as perceived by the naked eye. Only 20% of the identified studies compare the performance of various colour channels (Fig. 2a). For instance, several studies chose a channel with a bandwidth that matches the reflected colour of the assay [[40], [41], [42]]. Others however chose a channel with a bandwidth that matches the absorbed colour instead [31]. Thus, the B channel of RGB is used for assays with a variation in red [31] and blue intensity alike [42]. Moreover, both assays showed good linear ranges and LODs (linear range of 10–2000 μM for Cu^2+^ containing pesticide detection; LOD 0.795 μM [31] and linear range of 0.1 μM–1 mM for the pesticide thiram; LODs 0.1 μM [42]). This is interesting because the B channel was found to outperform the R channel for the quantification of both red and blue intensity based assays in a recent study performed by our research group [25]. Moreover, other studies comparing RGB channel performance equally identify different optimum channel choices (R [26] or B [34]) for blue intensity variations. Both studies compared channel performance of all three RGB channels and showed sensitive detection limits for their respective targets (0.2 ppm for formaldehyde detection in ginger and ginseng [34] and 1.51 U/mL alkaline phosphatase in milk [26]; see Table 1 for more detail). This underpins the importance of RGB channel performance comparisons during assay development. The apparent lack of correlation between assay colour and optimum channel choice may be attributed to metamerism causing limited correlation between perceived colour and spectral variance related to the analytical signal. Thus, measuring the spectral reflectance of the assay to determine in which bandwidth the variation of the analytical signal falls may be prudent [16] (Fig. 3b). Moreover, in this manner, it can be determined if blending of bandwidths, an option used in various studies (Fig. 2b inset; Table 2), should be considered. Worthy to notice is that in the same study [16], video recording of the assay was used instead of photo capturing, enabling the dynamic recording of the tested enzymatic reaction. In this way, an internal quality system is able to reject spurious measurements, based on principal components analysis (PCA). Finally, sensitive detection (LOD 50 ng.mL^−1^) of the pesticide carbofuran was achieved in apple matrix with only ~ 0.30 €/device material costs.

**Table 2:** An overview of SBDs used in the food safety field focusing on the performed image analysis. More publications can be found in the supplementary materials in excel spreadsheet form.

Target analyte	Matrix	Sensor type	Performed image analysis	Optimum space	Hardware	Software	# Phones	Ref
*Escherichia coli* and *Enterococcus* species	lettuce	liquid assay (enzymatic substrate conversion)	grayscale intensity from B	NC	box and flashlight (flash adapted)	ImageJ, no app	1	[35]
mercury (II)	tap water, serum	nanosheet with nanozyme activity (TMB conversion)	various RGB ratios	(G + B)/2R	no hardware, flash not specified	commercial App (colour assist)	1	[55]
iron	meat and liver	chromogenic assays (1,10-phenanthroline, 2,4,6-tris(2-pyridyl)-striazine, salicylate) for iron ions	RGB, Hunter-LAB, CIE-Lab, CIE-Luv, CIE-LCh, HSV, HSL ΔELab and ΔELuv	ΔELab and ΔELuv	box, white paper used as diffuser. Illumination with TL lamp	ImageJ, no app	1	[32]
benzoic acid	21 commercial food samples	μPAD using Janovsky reaction	sum of R and B channels	NC	box with LED and CMOS camera and additional hardware	in-house developed app	1	[56]
gluten, cow milk, pH	milk, cheese, soil	lateral flow devices, ELISA, nanoparticles on filterpaper	channels of RGB, HSV, LAB and weighed RGB	R, G or B (depending assay)	no hardware compared with box, flash used	commercial app (RGB android)	6	[25]
mercury ions, ochratoxin A and Salmonella	tap water	upconversion nanoparticle functionalised aptamers in lateral flow device	grey scale intensity of separate RGB channels	NC	3D box with LED array, lens, dichroic mirror	ImageJ, no app	1	[57]
chloramphenicol	milk and chicken	ssDNA-modified gold nanoparticle aggregation assisted by lanthanum ions.	B/R ratio to measure assay change of red to blue	NC	no hardware, flash not specified	commercial app (Touch Color app)	1	[58]
cyanide	apricot seeds	paper-based diaminomalonitrile-based receptors for cyanide detection	R/G ratio for yellow to red colour change	NC	no hardware, flash not specified	commercial app (color assist)	1	[59]
malathion, paraoxon	drinking water	acetylcholine inhibition, paper-based	combining R/B ratio with greyscale thresholds and weighed summation	NC	no hardware, no flash	in-house developed app	1	[60]
okadaic acid, saxitoxin	shellfish (mussel)	indirect competitive immunoassay (ELISA) in 96 well plates	HSV and RGB channels	S (although B was similar)	box, wide angle lens, Electroluminescence illumination	in-house developed app (iOS APP)	1	[24]
malathion	tap water	acetylcholinesterase immobilized on cellulose powder with smartphone read-out	RGB and CMYK channels	R found optimum	box, 24 LED lights	commercial app (Adobe capture)	1	[61]
chlorpyrifos	fruit & vegetable wash water	lipase paper based device	RGB, HSV and YCbCr channels	Cb found optimum	two systems: one no box, one box with LED light	MATLAB no-app	1	[62]
*Escherichia coli*	drinking water	NP-mAb paper assay with printed QR code.	Barcode analysis from RGB	no comparison	no hardware, flash not specified	AR-app (QR codes)	1	[52]
melamine & chloramphenicol	milk	immune-chromatographic chip	Barcode analysis from RGB	no comparison	no hardware, barcode chip, flash not specified	AR app (QR codes)	1	[53]

### 4.3 Other colour spaces

Some studies suggest that converting RGB values to other colour spaces improves performance. For intensity-based assays channels, such as greyscale, L of LAB or S of HSV have been suggested, whilst channels describing chromaticity (H of HSV, Cb of YCbCr) have been used for assays based on colour change (supplementary Table 1). However, the proportion of studies utilising these colour spaces is relatively low (Fig. 2b) and the few comparison studies which compared the performance of these alternative colour spaces with RGB show varying performance (Table 2). This may be because the raw data is inevitably in RGB and mathematical conversion to other colour spaces may cause the introduction of additional error. Another option is combining colour channels of various colour spaces into novel channel combinations to optimise the quantification of assay specific changes. This method has been successfully used for pH quantification in soil (mean average error 1.31 ± 0.02%; linear regression R^2^ = 0.997) and the detection of goat milk adulteration with cow milk (mean average error 36 ± 6%; linear regression R^2^ = 0.97) [43].

### 4.4 Illumination options

Another issue for smartphone based colorimetric assays is illumination. Approximately 55% of the studies analysed report the use of a light-shielding box to avoid measurement error due to background illumination variation. Approximately 45% report on direct measurements without a box (Fig. 2a). These studies use signal-to-background ratios [26,44], signal-to-control line ratios [20,21] or background subtraction [45] to limit the effect of background illumination. Interestingly, similar background corrections are equally used to limit illumination variation in a box [30,32,35] (see table one for details on analytical parameters). These studies utilise SBDs for the quantification of vastly different targets (from allergens [20,21], to enzymes [26,44], marine toxin [30] and pathogens [35]). This makes it difficult to compare performances and link those differences to the applied image analyses strategies since many other factors equally can affect performance. This being said, correlation coefficients for the studies using a light-shielding box (0.954 in Ref. [30], >0.99 in Refs. [32,35]) are similar to the correlation coefficients reported for the devices that did not use a light-shielding box (0.999 in Ref. [44] and 0.98 in Ref. [45]). Unfortunately no R^2^ values were given in Refs. [20,21]. However, both works showed that detection limits under 3 ppm were achievable thus equally showing good performance for allergen detection. Thus, the main advantage of a light-box may be to shield-off high intensity outdoor light and not limit error caused by background illumination variations in room light conditions. However, 80% of the studies that did not utilise a box gave no specific information regarding the use of the phones' flashlight (Fig. 2a), complicating reproduction. This was not the case for illumination used within light-shielding boxes where lighting parameters were clearly specified in each article (Fig. 2c). Light emitting diodes (LEDs), utilising additional batteries for power [23,46] or powered through the smartphones USB port [47], were the most common illumination source (~63%). This is most likely due to the low price and energy consumption of LEDs. LEDs were used for various SBDs including nanomaterial labelled lateral flow and fluorescence-based assays several of which showed good sensitivity and selectivity and R^2^ values above 0.98 (supplementary Table 1). Alternatively, using the smartphone flashlight instead has equally been reported [16,35] and constitutes an interesting alternative because it omits the use of additional parts. Notably, Adkins et al.*,* reports very good R^2^ values (>0.99) and an RSD that was reduced from 28% to 9.2% as well as a ~2 fold increase in sensitivity by adjusting the brightness of the flashlight [35]. This may be an interesting solution to overcome the issue of brightness saturation or inconsistent flash intensity. Another alternative light source is electroluminescence (EL). This illumination source is a cost-competitive light source and a Lambertian radiator, which provides a more constant and diffuse light source as spot-based LED light. This illumination source was successfully used by Su et al.*,* for the quantification of marine toxins (okadaic acid and saxitoxin) with commercial ELISA assays [24] and a cell viability kit [30]. Both works showed good sensitivity with LODs well below the maximum regulatory limits (MRL) in the EU for these toxins (Table 1). Especially the method reported in Ref. [24] was very sensitive (LOD for saxitoxin 0.0092 μg.L^−1^; LOD for okadaic acid 0.0864 μg.L^−1^). That is four orders of magnitude below MRL for saxitoxin and one order of magnitude below MRL for okadaic acid. Moreover, the RSD was shown to be only between 5 and 10%. This further shows the potential of smartphone-based ELISA quantification.

### 4.5 Other hardware

Other commonly used hardware are optical filters for fluorescent assays (19%) and additional lenses (23%) (Fig. 2b). Using this additional hardware makes fluorescent devices more expensive as colorimetric alternatives, but more apt for detection in strongly coloured matrices. Perhaps the most extensively modified smartphone based device was reported by Cheng et al., [22]. This device uses a fluorescent (quantum dots) aptamer-based lateral flow assay (R^2^ > 0.98) for the sensitive detection of pesticides in vegetables and fruits (LOD for chlorpyrifos 0.73 ng.mL^−1^, LOD for diazinon 6.7 ng.mL^−1^, LOD for malathion 0.74 ng.mL^−1^). The 3D printed fluorescent microscope consisted of a smartphone holder, additional lens, a CMOS sensor, diffraction grating, an optical filter and a laser (Fig. 3c). Although quite bulky, the device was able to convert RGB values to wavelengths (0.2 nm per pixel) utilising laser calibration, and thus could go beyond the 8-bit dynamic range and channel related bandwidth limitations of standard smartphone-based image analysis. Additional hardware, such as optical filters and lenses for fluorescent detection, has been avoided in other studies by utilising UV excitable labels and UV-LEDs, for example for the detection of phenols and polyphenols in wine samples [47]. This device (Fig. 3d) is especially interesting as it uses quantum dots for pesticide residue detection (as reported in Ref. [22]) but requires much less additional hardware while equally achieving good R^2^ values (>0.98). This being said, LODs for paraoxon, 4-nitrophenol and quercetin are in the ~5–10 μg.L^−1^ range, which is three orders of magnitude less sensitive as the method reported in Ref. [22]. In other studies, UV-excitable fluorescence enhancement is caused by the complexation of the target compound (tetracycline) with a lanthanide [29] or UV excitable fluorescence of the target compound itself (aflatoxin B1) [48]. Notably, both systems equally showed good R^2^ values (>0.98) and, for [29], outstanding RSD values (<5%). However, LODs were again in the ~5–10 μg.L^−1^ range showing very low limits of detection may be hard to achieve with these methods.

### 4.6 Sensing with the ambient light sensor

Some studies try to increase assay consistency by utilising the smartphone ambient light sensor (ALS). This method has been successfully used for the detection of zearalenone in cornflour by ELISA (R^2^ > 0.98; LOD 2.12 ng.mL^−1^) [49] and was reviewed recently [6]. A scheme depicting the device reported in Refs. [49] is reproduced in Fig. 3e. Error caused by reflection and background illumination is limited because transmitted light intensity is measured directly. However, this is equally the disadvantage of the method since it is not adapted to reflectance measurements from assays based on dry chemistry. Additionally, ALS resolution on smartphones can be quite low (around 1 lux [50]). ALS with better resolution (under 0.005 lx) exists but is generally not integrated in smartphones but sold as separate, be it very compact, devices [51]. These sensors typically feature a 16-bit dynamic range thus offering a considerable gain in dynamic range. This being said, no studies were identified studying interphone variation when ALS is used and this will need to be investigated.

### 4.7 Software use

Regarding software used for image analysis (Fig. 2d), three major groups can be identified:

(i) Reports on SBDs fitted with an in-house developed app designed for the quantification of the particular assay (32%).
(ii) Reports on SBDs that use an existing commercial colour app for colour quantification (25%) or QR code reading (5%).
(iii) Reports on SBDs that utilise PC-based software to perform off-phone image analyses (34%).

Group (i) constitutes SBDs that allow direct quantification of the target analyte with the phone without any additional data treatment, thus constituting a high technology readiness level. That being said, the data treatment performed by these devices is not always described in detail and the developed apps are not publicly accessible. This limits the possibility to reproduce the studies or further improve the developed software for other SBDs. Group (iii) lies on the other end of the spectrum. Here, the phone is merely being used for image acquisition and data analyses is mainly performed with ImageJ (Fig. 2d). On the other hand, programming applications such as MATLAB or Python can be combined with automated data transfer and cloud computing to compliment the development of these SBDs. Of group (ii), most publications report on devices with a technology readiness level similar to group (iii), albeit that the colour channel values are extracted on the smartphone. The use of commercial augmented reality (AR) apps however is perhaps further advanced. Here, the assay is designed as a barcode, which is either disturbed or changed by target recognition. The altered QR code is then read by the app, which displays the result within seconds. Examples are (i) a SBD for pesticide (methyl parathion) detection in apples with a LOD of 200 ng.mL^−1^ [41], (ii) a SBD for *E. Coli* detection in drinking water with positive detection from 10^6^ CFUs (Fig. 3f) [52] and, (iii), a SBD for melamine & chloramphenicol detection in milk with a LOD of 30 ng.mL^−1^ for melamine and 6 ng mL^−1^ for chloramphenicol [53] (Table 2). The concept of utilising QR barcodes is powerful because it utilises existing software and can be exploited for analyses by end-users using a plethora of smartphone models. However, further validation of these devices is needed. For instance, only [53] conducted an interference study. Moreover, verification of inter-phone variation is lacking and is needed to complete the readiness of this technique.

## 5 Commercial smartphone-based assays

Commercialization of smartphone-based analysis enables consumers' involvement in food testing. This increases public awareness, allows increased testing and improves food security. To date, few commercial products applying smartphone technology for food safety have been released. R-biopharm is a pioneer in smartphone-based colorimetrics for food safety analysis. The company has developed a SBD for mycotoxin detection (Aflatoxin, deoxynivalenol, zearalenon, fumonisin and fusarium toxins T2 and HT-2) aimed at industry and primary producers; the “RIDA®SMART APP Mycotoxin” [63]. The app achieves semi-quantitative results, much like an LFIA reader, whilst significantly decreasing analysis cost. The system uses a cover, containing a colour reference chart, and QR code, which is placed over the test strip prior to taking the image. Total assay time is only 10- to 15 min and requires no bulky laboratory equipment or laboratory expertise. To the best of the authors' understanding, the QR code and colour reference chart are used to calibrate the smartphone camera, identify the batch and standardize lighting conditions. The app provides a numerical read-out and enables real-time communication of the results (via e-mail), further simplifying the analytical procedure. On the downside, this processing system operates only in certain Android smartphones, again indicating the ultimate challenge of interphone operability that smartphone-based analysis needs to face.

To circumvent this issue, many companies avoid the smartphone camera and provide their own hardware. This means that the test only has to be optimised for one camera system, and not the myriad of available smartphone cameras. For example, Neogen Corporation sells many food analytic test kits that are compatible with their Raptor® system designed for non-technical staff in food companies. The Raptor® system is a small, portable, handheld camera device that can image LFIAs and report semi-quantitative results in real-time [64]. In another example, MyDx®, it is a commercially available handheld analyser that uses an AChE chip to screen carbamate and organophosphate pesticides and heavy metals in fruits, vegetables (OrganaDX; [65]) and water (AquaDX; [66]). Unfortunately, the LODs reported by the manufacturer were much higher than European MRLs. In both cases, the analyser is coupled to an in-house smartphone app able to attain one-click results. Similarly, Zeulab has designed the Test4all assay which combines a portable analyser with a smartphone app for the on-site antibiotic detection in milk [67]. Despite providing all the smartphone-related merits (portability, online connectivity etc.), there is not much information related to the bio-affinity part of the device as well as the test duration.

A major flaw with many of these solutions is that they are proprietary and only work with that company's own diagnostic assays. This is antithesis to the SBD philosophy, where the aim is to use one convenient device that everybody already has access to. Abingdon Health has developed a universal AppDx Smartphone Reader, an analysis system that functions very similarly to R-biopharm's system, but promises to work on more devices, including Apple iOS devices [68]. As another alternative, ChemBio Diagnostic Systems has developed the Cube-Reader. This very small and inexpensive handheld camera can also analyse and report LFIA results, however, the Cube-Reader stands out in that is extremely adaptable. The Cube-Reader can be programmed to work with any LFIA and ChemBio licenses the technology to other groups and researchers to use with their own tests [69]. Scienion AG is developing a similar universal analysis standard called the SciReader LF1. Uniquely, the SciReader LF1 can monitor assay development in real-time and report assay kinetics directly to the user's smartphone. Because the system does not rely on the smartphone camera, it can be used in conjunction with any device. (Personal correspondence).

The SCAN4CHEM APP [70] is a free application developed by the EU LIFE project allowing consumers to easily request product information, included in a database, regarding the presence of substances of very high concern (SVHC) by scanning the product barcode. SVHC includes 161 different compounds with proven carcinogenicity, persistence or bioaccumulation and manufacturers all over Europe are cordially invited to provide information on their products. In fact, SCAN4CHEM APP is developed not only for food but also cosmetics and chemicals used in households. Considering that SCAN4CHEM APP does not include any bioanalytical step and requires just barcode scanning, it is at the current stage the most realistic and feasible approach to inform the public about food safety. However, in this way, consumers are not truly involved in the food testing. This significant merit of smartphone-based analysis is an important objective in the EU-funded Horizon 2020 projects, FoodSmartphone [71] and PhasmaFood [72]. FoodSmartphone is focused on the development of bioanalytical assays with potential for user friendly smartphone based detection of allergens [20,21,25,43] and major food contaminants (e.g. marine toxins [73] and pesticides [16,74]). PhasmaFood aims to achieve on-site food quality testing developing a miniaturized smart sensor based on spectroscopy principles for example ultra-violet (UV) or infrared (IR) spectroscopy [[75], [76], [77]]. Specifically, miniaturized- or smartphone-spectral analysers [78] have emerged as a powerful technology to analyse food composition which can be extremely useful in cases of food adulteration or dietary information.

## 6 Conclusions

SBDs have been introduced in the food safety field as an alternative approach to traditional chemical assays, which are strictly performed under laboratory conditions by experts. Undoubtedly, smartphone-based assays have a strong potential to revolutionize the current food safety scheme in terms of on-site testing, citizens implementation and by significantly increasing the number of tested samples with a reduced cost. Importantly, an impressive number of novel SBDs have been reported in recent years; 47 of the 56 reviewed articles were dated between 2017 and 2020. Nevertheless, the application of SBDs for food contaminants detection is still in an early stage when considering the plethora of approaches that have been revealed in this systematic review, both on bioassay performance and image analysis.

Regarding method validation, it was found that only two out of 56 articles included all key parameters for analytical performance. To advance the field and develop SBDs that can be directly used by consumers and primary producers, it is necessary to change this trend and improve the technology readiness level of SBDs for food safety analysis. This is also reflected by the limited number of commercially available SBDs in the food safety sector. Established guidelines on screening methods (outlined in Ref. [2]) can be followed to better characterise SBDs.

In terms of image analysis, there is no consensus regarding an optimal and universal image processing workflow. Channel use is justified in various manners and the performance of a given channel of RGB space is not always compared with the remaining two. Moreover, experimental verification of the bandwidth of the analytical signal is rarely performed and this is easy to change. We recommend performing reflectance or absorbance spectroscopy for membrane and liquid based systems, respectively, to determine the useful analytical signal and corresponding bandwidth. If this is not possible, a “test and error” approach should be followed, evaluating at least all single RGB channels and, if needed, several channel combinations (additions or ratios) to find the optimum analytical signal. Furthermore, additional research to investigate the benefits of using alternative colour spaces should be done, as these results are inconclusive.

The interphone variation and the need to use calibration curves for each individual phone are great bottlenecks. Potential solutions can be testing more individual phone models and using standardised camera settings (ISO value, exposure, zoom) by using apps, for example the OpenCamera app for Androids. Utilising standardised illumination may equally be beneficial to limit inter-phone variance. There is also controversy related to the use of auxiliary parts to standardize lighting conditions during image data acquisition. The use of such equipment may be unnecessary if proper background correction is performed and one does not measure in direct sunlight. However, more experimental data is necessary to better understand this.

Finally, it was observed that more than half of the developed SBDs did not provide software enabling automated data analysis on the smartphone to provide an easy-to-use interface for the end-user. Thus, more effort needs to be directed to software development for SBDs.

## 7 Perspective

The main advantage of SBDs is the potential to transfer this technology to the consumer for at-home analysis. It is considered possible to accomplish this in the future with some considerations. The current main issues are the minimisation of inter-phone variation, optimising sample extraction protocols and integration of SBD software into operational apps while limiting the use of auxiliary parts. Fortunately, several strategies to resolve these issues are showing promising results. However, the solutions need to be combined effectively and the new devices validated in inter-laboratory validation studies. Once these steps are completed commercialization may enable consumers to purchase ready-to-use devices without the need to obtain reagents from commercial scientific sources.

Lastly, a thorough understanding of the limits of such devices (mainly inevitability of some false positive results due to the high number of tests performed if end-user based commercial uptake is accomplished) by the public is necessary to avoid misinterpretation. Although this final issue is substantial, it may be overcome through adequate communication to the public.

Overall, SBD technology still has some road ahead of it before it can be used for large scale food safety analysis by the consumer. This being said, promising solutions to the outlined issues exist and the field is advancing rapidly with several SBDs already available commercially. Thus, perhaps the main question remaining is when, not if, these devices will be commonly seen in restaurants, markets and on the farm.
