# Supplementary material for: Smartphone-based optical assays in the food safety field
Source: Trends Analyt Chem. 2020 Aug;129:115934. doi: 10.1016/j.trac.2020.115934 (PMC7457721; doi:10.1016/j.trac.2020.115934)
Supplement: Multimedia component 2 [file mmc2.docx]

**Supplementary information: Smartphone-based optical assays in the food safety field**

J.L.D. Nelis^a+*^, A.S. Tsagkaris^b+^, M. J. Dillon^a^, J. Hajslova^b^,

and C.T. Elliott^a^

1. Institute for Global Food Security, School of Biological Sciences, Queen’s University, 19 Chlorine Gardens, Belfast, BT9 5DL, United Kingdom.
2. Department of Food Analysis and Nutrition, Faculty of Food and Biochemical Technology, University of Chemistry and Technology Prague, Technická 5, 166 28 Prague 6 – Dejvice, Prague, Czech Republic.

*: corresponding author.

J.Nelis@qub.ac.uk

+: authors equally contributed

# Methods

The following keyword search term was used in the Scopus database to identify optical SBDs in the food safety field:

((("Smartphone" AND ("colorimetric" OR "optical" OR "lateral flow" OR "ELISA" OR "paper based" OR "flow through" OR "lab on a chip" OR "scanner")) AND ("food" OR "feed" OR "pesticides" OR "mycotoxins" OR "marine toxins" OR "pathogens" or "microcystin")))

Reviews, conference papers, and articles not written in English were automatically excluded leaving a total of 127 articles. Of these 14 articles were excluded upon title and abstract analyses because they were clearly out of scope. The eligibility of the remaining 113 articles was then analysed by verifying the inclusion of the following criteria in the full text.

- The article was in English and was not a review, conference paper or project description
- The article focused on a food safety related issue and not on any other field (including food quality/adulteration).
- The smartphone was used as the analytical detector of the developed assay.
- The ability to adequately detect the target analyte with the SBD in its matrix was verified.

Fifty-six articles fitted the given criteria and were thus included in this review. Next, a spreadsheet (Supplementary Table 1) was built, listing the analytical parameters of the identified SBDs as well as critical information regarding the image analysis performed and the hardware and software used. An overview of the selection process, exclusion (with reasons), and key parameters listed in the spreadsheet is shown in Fig 1.

Regarding commercial SBDs, the review process was more challenging as most of the information was not published in scientific journals beyond, in a few cases, early stage prototypes. Consequently, the search was based on using cross referencing, a recently published work from our group on commercial assays [1] and manual Google searches using a large variety of keywords.


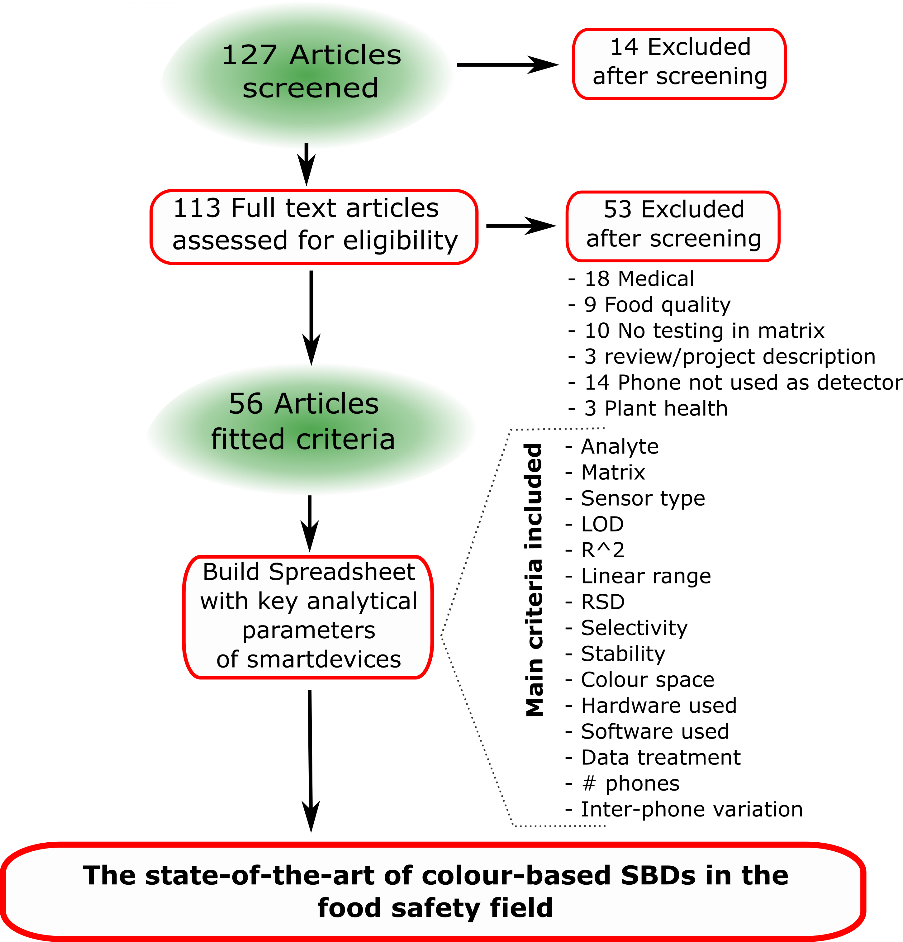


**Fig.1** Workflow of the selection process and information collection used.
